# A Cross‐Sectional Study to Determine *Candida* spp. Carriage in Libyan Patients With Type 2 Diabetes

**DOI:** 10.1155/bmri/5379778

**Published:** 2026-01-22

**Authors:** Mustafa H. M. Esmaio, Pedro M. D. S. Abrantes, Charlene W. J. Africa

**Affiliations:** ^1^ Maternal Endogenous Infections Studies (MEnIS) Research Laboratories, Department of Medical Biosciences, University of the Western Cape, Bellville, South Africa, uwc.ac.za

**Keywords:** antimicrobial surveillance, *Candida* prevalence, diabetes mellitus Type 2, drug resistance

## Abstract

The risk for *Candida* infections in patients with diabetes mellitus (DM) is three times higher than in the general population. Despite a DM prevalence of 15.8% reported in Libya (2024), the laboratory identification and susceptibility testing of fungal infections in patients with DM are not routinely performed. This cross‐sectional study investigated the prevalence and antifungal drug resistance patterns of *Candida* species in the oral mucosa of Libyan patients with Type 2 DM. Oral samples were collected with a sterile cotton swab, and 182 *Candida* isolates were phenotypically identified using the API ID 32 C and VITEK 2 Compact systems. Isolates were screened for their susceptibility to fluconazole and five other antifungals using disk diffusion and VITEK AST‐YS07 cards. Statistically significant associations were found between *Candida* carriage and clinical presentation (*p* = 0.032), denture wearing (*p* = 0.025) and sex (*p* = 0.012). Although *Candida albicans* was the predominant species isolated (37.4%), the majority of isolates comprised non‐albicans *Candida* (NAC). *Candida humicola* and *Candida dubliniensis* coexisted with other *Candida* species. Most *Candida* species showed susceptibility or dose‐dependent susceptibility (DDS) to fluconazole with low resistance to the other antifungal drugs. *Candida glabrata*, *Candida guilliermondii* and *Candida krusei* were resistant to fluconazole, and multidrug resistance was observed in some *C. albicans*, *C. dubliniensis* and *C. krusei* isolates. *Candida membranifaciens and Candida parapsilosis* showed either DDS or resistance to fluconazole. The emerging resistance to second‐line antifungals requires the establishment of routine *Candida* identification and antifungal susceptibility testing to guide species‐specific treatment.

## 1. Introduction

Diabetes mellitus (DM) comprises a group of disorders characterised by elevated levels of glucose in the blood and serious life‐threatening health conditions affecting the heart, eyes, kidneys and nerves [1]. As one of the most significant chronic diseases worldwide, its prevalence in 2022 was estimated at 830 million people [1]. Population growth, ageing, urbanisation and the rising incidence of obesity and physical inactivity have contributed to this increase, with > 3.4 million people estimated to have died as a result of DM in 2024 [2].

Type 2 diabetes mellitus (T2DM) or noninsulin‐dependent DM is associated with a sedentary and western lifestyle and represents approximately 90% of diagnosed DM cases, affecting mostly people who are middle‐aged and obese [3]. In Libya, the prevalence of DM stood at 15.8% in 2024, with this number estimated to increase to 1 million by 2050 [2]. These statistics are compounded by the obesity epidemic in Libya [4, 5]. Apart from obesity, risk factors such as family history of DM, hypertension and microvascular complications necessitate a Libyan national policy for the surveillance, prevention and control of diabetes and its complications [6], and one such complication is candidiasis, caused by several species of the genus *Candida*.

*Candida* exists in the human host as commensal organisms but may cause opportunistic fungal infections when the host homeostasis is disturbed. *Candida* infections of the oral cavity are common in patients with immunosuppression [7], and because of increased salivary glucose and xerostomia [8, 9], patients with DM are three times more likely to have *Candida* infections compared to the general population [10]. *Candida* pathogenicity is enhanced by the heightened availability of *Candida* receptors [11] in patients with DM, as well as the enzyme activity and biofilm formation of *Candida* strains [12].

*Candida albicans* and *Candidozyma auris* are listed as critical priority group members in the WHO Fungal Priority Pathogens List [13], with *Candida tropicalis*, *Candida parapsilosis* and *Candida glabrata* listed in the high‐priority group of fungal pathogens [13]. Therefore, regular surveillance and public health interventions are imperative. Increasing reports of antifungal resistance and the frequent finding of increased non‐albicans *Candida* (NAC) species highlight the need for regular epidemiologic studies to identify new species prevalence and antifungal profiles that may alter the diagnosis and treatment of candidiasis. This is particularly relevant on the African continent, where resources are often limited and exceeded by treatment needs [14].

Multidrug‐resistant candidiasis has increasingly been associated with hospital settings. Reports from several hospitals in Africa reveal differences in *Candida* species prevalence and antifungal drug resistance. In South Africa, the epidemiology of *Candida* species has changed over the past 20 years, with the prevalent species shifting from *C. albicans* to *C. parapsilosis* and outbreaks of *C. auris* reported in a tertiary hospital setting [15–17]. *C. auris* has also been identified in patients from distinct healthcare facilities in Nigeria [18]. In Algeria, environmental and patient screening in seven hospitals in Algiers reported that *C. tropicalis* was the most prevalent, followed by *C. parapsilosis*, *C. albicans* and *C. glabrata* [19], with seven cases of *C. auris* infection reported from an intensive care unit (ICU) outbreak in 2022 [20]. Studies from two hospitals in Cairo, Egypt, reported *C. albicans* prevalence, followed by *C. tropicalis* and *C. parapsilosis*, with no *C. glabrata*. Less common NAC species were reported in the order of *Candida lusitaniae*, *Candida utilis* and *Candida kefyr* [21], while among patients in a tertiary hospital in Cairo, > 80% of multidrug‐resistant *Candida* isolates were identified as *C. auris* [22]. *C. albicans* was also the most predominant species reported from Tunisia, followed by *C. parapsilosis*, *C. tropicalis* and *C. glabrata*, with an overall fluconazole resistance of 6.7% [23]. Fluconazole resistance has been associated with its widespread and indiscriminate use in Africa, with only 12 African countries having access to echinocandins [24]. In this challenging and dynamic epidemiological landscape, Libya faces its own challenges, and regular monitoring of antimicrobial‐resistant bacterial and fungal species is required.

The laboratory identification and susceptibility testing of fungal infections is not commonly performed in Libya, with patients treated empirically according to their clinical symptoms. Suspected oral *Candida* infections are treated with nystatin suspension or fluconazole, the most widely prescribed antifungal drug for localised and disseminated candidiasis [25, 26], with no accurate identification or susceptibility testing performed. Hence, the treatment of seemingly similar *Candida* infections is not always done with the appropriate class of antifungal. In addition, the antifungal armamentarium in Libya is limited, with only lipid‐associated amphotericin B, fluconazole, voriconazole and itraconazole being available [27, 28].

A review of the burden of invasive fungal infections in Arabic countries showed an absence of published data on the prevalence of invasive *Candida* infection in Libya [29]. Although recent reports of *Candida* prevalence in Libya pertaining to vaginal candidiasis and DM have emerged [30, 31], there is a paucity of studies pertaining to oral *Candida* carriage in Libyan patients, particularly in those with DM.

This cross‐sectional study was aimed at investigating the prevalence and antifungal drug resistance patterns of *Candida* species isolated from the oral mucosa of Libyan patients with T2DM.

## 2. Materials and Methods

### 2.1. Ethical Considerations

Ethical clearance and authorisation for sample collection for this project were granted by the Ethics Committee at the University of Western Cape, South Africa (Ethics Clearance 14/3/1).

Prior to sample collection, the reasons for and nature of the study were explained to the patients who willingly consented to participate by signing a consent form. The study complied with the Declaration of Helsinki and its amendments [32].

### 2.2. Sample Collection

Three hundred thirty (330) patients with T2DM from the Misrata Diabetes Centre, Misrata, Northwest Libya, participated in this study. Samples were collected by scraping the patients′ oral mucosa and tongue with a sterile cotton swab. No information was gathered regarding oral hygiene practices or feeding prior to sample collection.

### 2.3. Inclusion and Exclusion Criteria

Only adult patients (24–95‐year‐olds) previously diagnosed with T2DM were selected. Patients who had been on antifungal therapy 2 weeks prior to sample collection were excluded from the study.

### 2.4. Isolation and Identification of *Candida* Species

The collected oral swabs were taken to the medical microbiology laboratories at Misrata Central Hospital, cultured on Sabouraud dextrose agar (SDA, Cat. No. 84088, Merck, Darmstadt, Germany) and incubated at 37°C for 24 h. Agar plates showing no growth were reincubated for an additional 24 h before being discarded as negative. All isolated *Candida* strains were stored at −80°C in Pro‐Lab Microbank microbial preservation vials (Cat. No. PL.170/M, Pro‐Lab, Canada) and SDA slants in 2.5 mL Eppendorf tubes, allowing them to be revived as and when needed. Samples were transported from Libya in these frozen preservation vials to the Maternal Endogenous Infections Studies (MenIS) Research Laboratories at the Department of Medical Biosciences, University of the Western Cape, South Africa, for characterisation.

Phenotypic screening for *Candida* species was achieved using conventional methods (microscopy, gram staining and the germ tube test). Isolates were presumptively identified to species level by streaking pure colonies on Fluka chromogenic *Candida* identification agar (Cat. No. 94382, Merck, Darmstadt, Germany) and Oxoid chromogenic *Candida* agar (Cat. No. CM1002A, Oxoid, Hampshire, United Kingdom), according to the manufacturers′ instructions. Species identification was confirmed using the VITEK 2 Compact system (bioMérieux, Marcy l′Étoile, France), an automated miniaturised microdilution system for routine clinical laboratory identification, and sensitivity testing was achieved with the YST test kit for yeasts (Cat. No. 21343, bioMérieux, Marcy l′Étoile, France). Isolates that could not be identified with the VITEK system were identified by biochemical testing using the API ID 32 C system (bioMérieux, Marcy l′Étoile, France), which conducts 29 assimilation tests and includes a colorimetric test for easy interpretation of results.

Nine *Candida* type strains were used as quality control organisms for the species identification and antifungal drug susceptibility testing, namely, *C. albicans* (ATCC 90028 and NCPF 3281), *C. tropicalis* (ATCC 950), *Candida dubliniensis* (NCPF 3949a), *C. glabrata* (ATCC 26512), *Candida krusei* (ATCC 2159), *C. parapsilosis* (ATCC 22019), *C. kefyr* (ATCC 4135) and *C. lusitaniae* (ATCC 3449). The nine *Candida* type strains were matched to the exact identification with the API ID 32 C and VITEK 2 Compact systems.

### 2.5. Antifungal Drug Susceptibility Testing

#### 2.5.1. Agar Disk Diffusion

Isolates were screened for their susceptibility to fluconazole using disk diffusion by placing 25 *μ*g fluconazole disks (Cat. No. X7148, Oxoid, Hampshire, United Kingdom) on yeast nitrogen base agar with glucose (YNBG) plates (Cat. No. 239210, Becton, Dickinson and Company, Swindon, United Kingdom) previously spread with a 0.5 McFarland standard suspension of the organisms (using a sterile cotton swab) and incubating at 37°C for 24 h. After incubation, the zones of inhibition around the fluconazole disks were measured from the edge of the disk to the edge of the susceptibility area, and the presence of microcolonies within the susceptibility zone noted and scored as previously described [33]. Samples with susceptibility areas < 7 mm with the presence of microcolonies were regarded as resistant, as well as samples with a microcolony score of > 2, while samples with susceptibility areas > 12 mm and a microcolony score of ≤ 2 were regarded as susceptible to fluconazole. Strains with a susceptibility area ranging from 7 to 12 mm were regarded as intermediate (or dose‐dependent) strains. Tests were done in triplicate. Microcolony and outer growth areas of random samples were stained and observed microscopically (Nikon SE, 1000x) for *Candida* species confirmation. These were subsequently grown on Oxoid and Fluka chromogenic *Candida* agars to confirm the species.

#### 2.5.2. VITEK 2 Susceptibility Testing

VITEK AST‐YS07 cards (Cat. No. 414967, bioMérieux, Marcy l′Étoile, France) were used to confirm the species and antimicrobial sensitivity testing of the isolates. The susceptibility to six antifungal drugs was tested, namely, amphotericin B, caspofungin, micafungin, fluconazole, voriconazole and flucytosine.

Fresh 24‐h *Candida* subcultures were inoculated into sterile test tubes containing saline, and the density was adjusted to a 1.80–2.20 McFarland standard using the supplied VITEK nephelometer. A standardised concentration of the suspension in 0.45% saline was used to rehydrate the antifungal medium within the card according to the manufacturer′s instructions. Minimum inhibitory concentration (MIC) values were determined for each antifungal contained on the card according to the most updated Clinical and Laboratory Standards Institute (CLSI) interpretation given by the VITEK system. MICs were defined as the lowest concentrations that inhibited growth at 100%.

### 2.6. Statistical Analysis

Numbers (percentage) used to express qualitative data and quantitative data are presented by mean and standard deviation. Chi‐square was used to demonstrate the association between *Candida* species and patient data using the SPSS statistics software (SPSS Inc., Chicago, Illinois, United States) Version 24.0. *p* < 0.05 was considered significant.

## 3. Results

Of the 330 patients, 170 (51.5%) had *Candida* carriage, of whom 13 (7.6%) were diagnosed with active candidiasis and the remaining 92.3% with subclinical presentation. In total, 182 isolates were collected (12 patients carried two *Candida* species).

Of those with *Candida* carriage, 56% were female and 60% wore dentures, with most *Candida*‐positive participants located in the age range of ≥ 35 years (Table 1). Statistically significant associations were found between *Candida* carriage and patient clinical presentation (clinical vs. subclinical candidiasis, *p* = 0.032), denture wearing (higher *Candida* carriage in denture wearers, *p* = 0.025) and sex (higher *Candida* carriage in females, *p* = 0.012). No significant associations were found with smoking and age distribution (Table 1).

**Table 1:** Patient variables associated with *Candida* carriage.

	***Candida* carriage**	**Dentures**	**Sex**		**Smoking**	**Age distribution (years)**		
			**M**	**F**		**15–34**	**35–64**	**≥ 65**
*Candida*	170 (51.5%)	60 (60.6%)	53 (42.4%)	116 (56.6%)	17 (38.6%)	3 (75%)	99 (48.5%)	67 (54.9%)
No *Candida*	160 (48.5%)	39 (39.4%)	72 (57.3%)	89 (43.4%)	27 (61.4%)	1 (25%)	105 (51.5%)	55 (45.1%)
Total	330 (100%)	99 (100%)	125 (100%)	205 (100%)	44 (100%)	4 (100%)	204 (100%)	122 (100%)
*p* value		0.025	0.012		0.073	0.339		

### 3.1. Frequency of *Candida* Species Isolated

**Figure 1:**
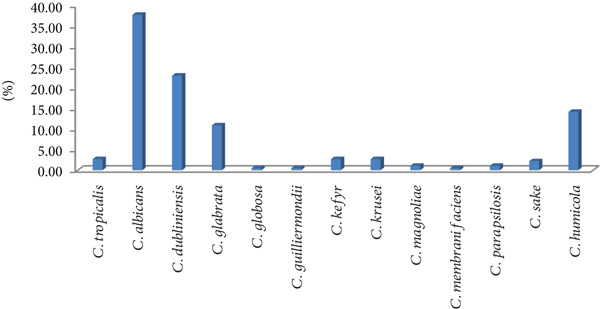
Distribution of *Candida* species identified by VITEK and API ID 32 C.

Sixty‐eight of the 182 isolates (37.4%) were identified as *C. albicans* and 114 (62.6%) as NAC, including 42 (23%) *C. dubliniensis*, 26 (14.28%) *Candida humicola*, 20 (10.9%) *C. glabrata* (now known as *Nakaseomyces glabratus*, but to facilitate comparisons with other studies, we will retain the name *C. glabrata* within this manuscript), five (2.74%) *C. krusei* (now called *Pichia kudriavzevii* but referred to as *C. krusei* in this paper), five (2.74%) *C. tropicalis*, five (2.74%) *C. kefyr*, four (2.2%) *Candida sake*, two (1.1%) *C. parapsilosis*, two (1.1%) *Candida magnoliae*, one (0.55%) *Candida guilliermondii*, one (0.55%) *Candida globosa* and one (0.55%) *Candida membranifaciens* (Figure 1). None of the isolates were identified as *C. auris.*

### 3.2. Antifungal Drug Susceptibility

#### 3.2.1. Agar Disk Diffusion

One hundred fourteen (114), 31 and 37 of the 182 species were susceptible, intermediate (dose‐dependent susceptibility [DDS]) and resistant to fluconazole, respectively (Table 2). The majority of *C. albicans*, *C. dubliniensis*, *C. humicola*, *C. kefyr*, *C. magnoliae*, *C. sake* and *C. tropicalis* strains showed susceptibility or DDS to fluconazole, while *C. glabrata, C. guilliermondii* and *C. krusei* were resistant. *C. membranifaciens* and *C. parapsilosis* showed DDS or resistance to fluconazole.

**Table 2:** Fluconazole susceptibility using agar disk diffusion and VITEK.

***Candida* species**		**Susceptible,** **n** **(%)**	**Intermediate (dose-dependent susceptibility),** **n** **(%)**	**Resistant,** **n** **(%)**	**Total,** **n** **(%)**
*C. albicans*	ADD	52 (76.4)	12 (17.6)	4 (5.8)	68 (100)
	VITEK	64 (94.1)	2 (2.9)	2 (2.9)	68 (100)
*C. dubliniensis*	ADD	33 (78.6)	8 (19.0)	1 (2.4)	42 (100)
	VITEK	10 (76.9)	1 (7.7)	2 (15.3)	13 (100)
*C. glabrata*	ADD	0	0	20 (100)	20 (100)
	VITEK	0	20 (100)	0	20 (100)
*C. globosa*	ADD	1 (100)	0	0	1 (100)
	VITEK	ND	ND	ND	ND
*C. guilliermondii*	ADD	0	0	1 (100)	1 (100)
	VITEK	ND	ND	ND	ND
*C. humicola*	ADD	20 (76.9)	3 (11.5)	3 (11.5)	26 (100)
	VITEK	ND	ND	ND	ND
*C. kefyr*	ADD	2 (40)	3 (60)	0	5 (100)
	VITEK	1 (50)	1 (50)	0	2 (100)
*C. krusei*	ADD	0	0	5 (100)	5 (100)
	VITEK	0	0	5 (100)	5 (100)
*C. magnoliae*	ADD	2 (100)	0	0	2 (100)
	VITEK	ND	ND	ND	ND
*C. membranifaciens*	ADD	0	1 (100)	0	1 (100)
	VITEK	ND	ND	ND	ND
*C. parapsilosis*	ADD	0	1 (50)	1 (50)	2 (100)
	VITEK	1	0	0	1 (100)
*C. sake*	ADD	2 (50)	0	2 (50)	4 (100)
	VITEK	ND	ND	ND	ND
*C. tropicalis*	ADD	2 (40)	3 (60)	0	5 (100)
	VITEK	2 (50)	2 (50)	0	4 (100)
	**ADD**	114 (62.6)	31 (17.1)	37 (20.3)	**182 (100)**
	**VITEK**	78 (69)	26 (23)	9 (8.0)	**113 (100)**

*Note:* The bold data refers to the total numbers for ADD and VITEK.

Abbreviations: ADD, agar disk diffusion; ND, not detected (no interpretive breakpoints available).

#### 3.2.2. VITEK

Because of limited resources, not all the isolates were subjected to VITEK analysis. Only species for which interpretative breakpoints are available and species showing DDS or resistance to fluconazole using ADD were included. Thus, 142 isolates (with only 13/42 *C. dubliniensis* species) were tested using VITEK. Seventy‐eight (78) isolates were categorised as susceptible to fluconazole by the VITEK 2 Compact system (Table 2), with 35 confirmed as either DDS (*n* = 26) or resistant (*n* = 9).

Regarding the control type strains, the intrinsically resistant *C. glabrata* was categorised as resistant to fluconazole by ADD and DDS by the VITEK system, and *C. krusei* was categorised as resistant by both ADD and VITEK. Other type strains were mostly susceptible to fluconazole by ADD and VITEK, except for *C. tropicalis* and *C. kefyr*, which expressed DDS.

Three species for which no interpretative breakpoints have yet been established on VITEK showed moderate to high fluconazole MIC values: three *C. sake* isolates between 4 and 8 *μ*g/mL, one *C. membranifaciens* isolate with an MIC of 4 *μ*g/mL and one *C. guilliermondii* isolate with an MIC of 2 *μ*g/mL. Most of the *Candida* isolates demonstrated low resistance to the other antifungal drugs (Table 3).

**Table 3:** *Candida* species susceptibility on VITEK.

**Antifungal drugs**	**Interpretation**	***C. albicans* (** **n** = 68**)**	***C. glabrata* (** **n** = 20**)**	***C. dubliniensis* (** **n** = 13**)**	***C. krusei* (** **n** = 5**)**	***C. tropicalis* (** **n** = 4**)**	***C. kefyr* (** **n** = 2**)**	***C. parapsilosis* (** **n** = 1**)**
Amphotericin B	Susceptible	68	18	13	4	4	2	1
	Intermediate	0	0	0	0	0	0	0
	Resistant	0	2	0	1	0	0	0

5‐Flucytosine	Susceptible	68	20	12	0	4	2	1
	Intermediate	0	0	0	0	0	0	0
	Resistant	0	0	1	5	0	0	0

Caspofungin	Susceptible	68	0	13	5	4	2	1
	Intermediate	0	20	0	0	0	0	0
	Resistant	0	0	0	0	0	0	0

Micafungin	Susceptible	68	20	12	5	4	2	1
	Intermediate	0	0	1	0	0	0	0
	Resistant	0	0	0	0	0	0	0

Fluconazole	Susceptible	64	0	5	0	2	1	1
	Intermediate	2	20	0	0	2	1	0
	Resistant	2	0	3	5	0	0	0

Voriconazole	Susceptible	67	20	7	5	4	2	1
	Intermediate	0	0	0	0	0	0	0
	Resistant	1	0	1	0	0	0	0

*Note:* Only species for which interpretative breakpoints are available are shown. Intermediate = dose‐dependent susceptibility (DDS).

*C. humicola* (6/26 isolates) and 5/42 *C. dubliniensis* isolates coexisted with other *Candida* species in the oral cavity such as *C*. *albicans* (three isolates), *C. krusei*, *C. kefyr* (three isolates each) and *C. magnoliae* (Table 4).

**Table 4:** Data of patients from whom two *Candida* species were isolated.

**Species susceptibility**		**Species susceptibility**		**Sex**	**Age**	**Dentures**	**Presentation**
*C. humicola*	S	*C. kefyr*	I	F	65	No	Subclinical
	S	*C. kefyr*	I	M	76	No	Subclinical
	S	*C. krusei*	**R**	F	75	No	Candidiasis
	I	*C. dubliniensis*	I	M	62	No	Subclinical
	**R**	*C. tropicalis*	I	M	70	Yes	Candidiasis
	S	*C. albicans*	S	F	60	No	Subclinical

*C. dubliniensis*	I	*C. membranifaciens*	I	M	66	Yes	Subclinical
	S	*C. globosa*	S	F	62	Yes	Subclinical
	S	*C. parapsilosis*	I	F	58	No	Subclinical
	S	*C. albicans*	S	M	78	No	Subclinical

*C. albicans*	S	*C. glabrata*	**R**	M	54	Yes	Candidiasis

*C. magnoliae*	S	*C. kefyr*	I	F	71	Yes	Subclinical

*Note:* The bold letters refer to the resistant organisms (that were only identified in patients with candidiasis).

Abbreviations: DDS, dose‐dependent susceptibility; I, intermediate; R, resistant; S, susceptible.

In patients who had two species of *Candida*, candidiasis was only apparent when one of the species showed resistance to fluconazole. Two of the three patients with candidiasis and who harboured two *Candida* species were denture wearers (Table 4).

## 4. Discussion

Accurate identification and susceptibility testing of fungal species are increasingly important, especially in the face of emerging resistance to commonly dispensed drugs. Differences in the treatment guidelines between various geographical areas can result in the emergence of strains resistant to different classes of antifungal agents. Because *Candida* infections are commonly associated with T2DM, regular monitoring of species prevalence is essential to ensure adequate treatment planning. This study highlights the prevalence of *Candida* species and their susceptibility profiles in a cross‐section of patients with T2DM in Misrata, Libya.

As in the study by Mussi et al. [34], several patients in our study presented with subclinical infection despite *Candida* carriage. Among the factors that predispose to candidiasis in patients with DM, this study considered sex, age, species frequency and denture wearing. Statistically significant associations were found between *Candida* carriage and clinical presentation, sex and denture wearing, with no significant associations with smoking and age distribution. Published reports show contradictions regarding the association between sex and *Candida* carriage [10]. Some show no significant differences in *Candida* carriage between the sexes [35], and others report a higher prevalence in females [36–38]. This may be attributed to hormonal changes [39].

Epidemiology studies suggest that distinct species predominate in different regions and in different body sites. Recent studies of patients with DM focused mainly on samples from the vagina, foot ulcers, gastrointestinal/urinary tracts and the oral cavity. A Libyan study of vaginal, toe and nail swabs from patients with DM [31] reported *C. albicans* prevalence, followed by *C. tropicalis* and *C. glabrata*. Species isolated from DM foot ulcers reported the predominating species as *C. albicans* (75%), *C. lusitaniae* (8%) and *C. dubliniensis* (5%) in Kenya [40]; *C. parapsilosis* (71.5%) and *C. albicans* (14.3%) in Iran [41]; *C. albicans* (13.2%) and a predominance of NAC (58.4%) comprising *C. tropicalis*, *C. krusei*, *C. parapsilosis*, *Candida lipolytica*, *C. lusitaniae* and *C. guilliermondii* in Iraq [38] and *C. albicans* (71.4%), *C. tropicalis* (21.4%) and *C. glabrata* (7.1%) in Egypt [42]. In Uganda [43], samples from the gastrointestinal tract showed that *C. albicans* (62.16%) was the predominant species, followed by *C. glabrata* (18.92%), *C. tropicalis* (8.11%), *C. krusei* (5.41%) and *C. dubliniensis* (5.41%), while a Nigerian study of midstream urine samples of patients with DM [44] reported isolates of *C. albicans* (45.7%), *C. krusei* (28.6%), *C. glabrata* (20%) and *C. tropicalis* (5.7%).

In this study of samples from the oral cavity, *C. albicans* showed the highest prevalence (37.4%), followed by *C. dubliniensis* (23%) and *C. humicola* (14.3%), while a recent Libyan study of oral samples showed *C. albicans* (66%), *C. tropicalis* (13.6%) and *C. dubliniensis* (5.1%) predominating, along with other NAC species [45]. Oral isolates from Slovakia [46] also showed *C. albicans* predominating (61.9%), followed by *C. krusei* (14.3%), *Candida valida* (9.5%) and *C. glabrata*, *C. tropicalis* and *Candida intermedia* (4.8% each).

Interestingly, our study identified a wide variety of *Candida* species in Libya, supporting a reported worldwide shift from *C. albicans* only to NAC prevalence. However, a review of *Candida* carriage and infection in patients with DM revealed a predominance of *C. albicans* and *C. tropicalis*, with fewer NAC infections detected in patients with DM than without DM [10]. When looking at neighbouring countries, studies from Egypt [21, 47] and Tunisia [23, 48] also reported *C. albicans* as the dominant species detected in fungal infections, followed by *C. glabrata*.

Overall, our results showed a low prevalence of drug resistance for most species present in this population and are in accordance with a recent Libyan study [45] that reported susceptibility or intermediate susceptibility to most of the antifungals tested. Multidrug resistance was observed in a *C. albicans* isolate (resistant to fluconazole and voriconazole), a *C. dubliniensis* isolate (resistant to fluconazole, voriconazole and 5‐flucytosine) and five *C. krusei* isolates (all resistant to fluconazole and 5‐flucytosine, with one of them also resistant to amphotericin B). The high resistance levels of all *C. krusei* (*P. kudriavzevii*) isolates to fluconazole and the intermediate susceptibility seen in *C. glabrata* (*N. glabratus*) are to be expected, since these species are intrinsically resistant to this drug. However, the resistance of *C. krusei* to flucytosine and amphotericin B is a cause for concern. Echinocandins and amphotericin B are considered optional empirical treatments for azole‐resistant infections. All tested *C. glabrata* isolates showed susceptibility to micafungin and intermediate susceptibility to caspofungin, with two of these isolates being resistant to amphotericin B.

To the best of our knowledge, this is the first time that widespread cross‐resistance of the intrinsically fluconazole‐resistant *C. krusei* and *C. glabrata* to a pyrimidine analogue and an echinocandin, respectively, is reported in Libyan patients. However, this finding should be interpreted with caution, since caspofungin testing for *C. glabrata* was reported to be unreliable [49] and echinocandin susceptibility was better determined by the VITEK micafungin result. The discrepancies reported for caspofungin have been attributed to an echinocandin phenomenon in which fungal species are completely susceptible at low concentrations but may subsequently grow in higher concentrations of the echinocandin. Because of this variability, the CLSI recommends that intermediate or resistant caspofungin results should be confirmed with a micafungin or anidulafungin test [50]. The finding of more than one *Candida* species colonising 12 of the patients in our study could also influence the transmission of resistance traits between *Candida* species and their increased pathogenicity in the oral biofilm. Co‐colonisation of *Candida* species was associated with three cases of candidiasis where one of the species showed resistance to fluconazole. In all three cases, the resistant species were *C. glabrata*, *C. tropicalis* and *C. krusei*. Other co‐colonisers such as *C. dubliniensis* and *C. kefyr* showed intermediate susceptibility. This could signal the exchange of resistance genes and subsequent predisposition of the host to further fungal colonisation and infection. The combined growth of *Candida* species can result in increased biofilm formation [51], allowing for metabolic cooperation within the polymicrobial biofilm and protection from the external environment, immune responses and antifungals [52, 53]. The aforementioned species were also detected in oral samples from Mexican patients with mixed *Candida* infections involving two or three species [54]. Mixed oral candidiasis occurs in approximately 10% of patients with T2DM [54], underscoring the importance of species identification and susceptibility to ensure effective treatment.

Two of the three patients with candidiasis in our study and who harboured two *Candida* species were denture wearers. Denture‐wearing patients with DM are twice as likely to present with *Candida* colonisation than nondenture wearers [10] because of the potential for *Candida* to adhere to acrylic surfaces and form biofilms.

The VITEK 2 Compact system has the advantage of being fully automated and allowing both fungal species identification and MIC determination rapidly and simultaneously. It also yields similar results to the antimicrobial susceptibility testing procedures developed by the CLSI [50, 55] and is available in most clinical microbiology laboratories. However, its accuracy in species identification is sometimes questionable [56]. For example, our comparison of API and VITEK species identification agreed for most species, but *C. sake*, *C. globosa* and *C. membranifaciens* were not correctly identified by VITEK, and 26 isolates identified as *C. humicola* by API were categorised as the yeast‐like fungus *Stephanoascus ciferrii* on VITEK.

VITEK susceptibility appeared to be more sensitive than ADD in some cases, with intermediate/resistant reports by ADD showing as susceptible/intermediate with VITEK. Inaccuracies in *Candida* susceptibility have previously been reported [37, 47]. In a study comparing broth microdilution (BM) assays with VITEK, major errors were reported for *C. albicans* susceptibility to fluconazole and voriconazole (12.5% and 4.2%, respectively), with 5.3% very major errors and 10.5% minor errors found for *C. parapsilosis* and 33.3% minor errors observed in *C. tropicalis* for fluconazole [57].

Similarly, VITEK antimicrobial susceptibility assays have been described as unacceptable for colistin [58] and tigecycline susceptibility [59] in studies of enteropathic gram‐negative bacilli, with BM and ADD, respectively, showing more reliable results. A preference for ADD over VITEK for susceptibility testing of unusual Enterobacteriaceae species has been reported previously [60].

Testing of uncategorised species by VITEK may result in an unidentified result or a misidentification, and false susceptibility reports may be detrimental to patients if inappropriate therapy is administered. Newer techniques for species identification have been proposed to eliminate the error of misidentification [61, 62]. However, these techniques are costly and not readily available within most regions on the African continent.

This study has several limitations: (i) The *Candida* identification to species level relied on phenotypic and biochemical testing. In the absence of molecular identification methods and because VITEK had not yet been updated to identify *C. auris* [63], we may have missed the identification of this important species. (ii) Newly described or rarer species of *Candida* are not included in the VITEK YST database. This, along with differences in sample size for the ADD and VITEK assays and the inability to determine critical breakpoints for all the species tested, prevented an accurate calculation of categorical agreement or error rates between the two assays. (iii) The clinical diagnosis of oral candidiasis was crude and limited to the presence of white lesions on the tongue or inner cheeks to differentiate between clinical infection and a carrier state. (iv) Confounding factors that may have contributed to *Candida* colonisation, such as oral hygiene practices and the use of chronic medications, were not considered in the collection and analysis of the data. (v) This is a cross‐sectional study conducted in one region (single‐centre setting) with a limited sample size and did not include a control group of patients without DM. We were therefore unable to compare the prevalence of NAC in patients with and without DM. (vi) The paucity of current data regarding the burden of disease as well as management and treatment practices in this region limits the comparison of our study with others in Libya to recommend effective treatment strategies.

Although previously excluded from global antimicrobial resistant programmes [64, 65], the escalating antifungal resistance has sparked a series of studies on alternative strategies to inhibit or eradicate *Candida* biofilms, and they show promise for the development of new and improved treatment methods [35, 66–69]. The emerging resistance to second‐line antifungals observed in this study requires further investigation incorporating the routine identification of infection‐causing *Candida* species, their antifungal resistance gene expression, along with continuing surveillance and implementation of species‐specific treatment in conjunction with the most recent diagnostic and treatment recommendations and within the context of One Health [70, 71].

## Ethics Statement

Ethical clearance and authorisation for sample collection for this project were granted by the Ethics Committee at the University of Western Cape, South Africa (Ethics Clearance 14/3/1).

## Disclosure

All authors read and approved the final manuscript. The manuscript is based on the thesis by one of the authors [72].

## Conflicts of Interest

The authors declare no conflicts of interest.

## Author Contributions

M.H.M.E. performed the laboratory isolation, identification and drug susceptibility of the *Candida* isolates, analysed the data and prepared the first draft of the manuscript. P.M.D.S.A. participated in the study′s design and coordination and contributed to the writing and revision of the manuscript. C.W.J.A. conceptualized the study and participated in its design, coordination, writing and revision of the manuscript. M.H.M.E. and P.M.D.S.A. contributed equally to this paper.

## Funding

This study was funded by the Libyan Government (395/2013).

## Data Availability

Data is provided within the manuscript and is available from the corresponding author upon reasonable request.
